# Impact of Indoor Environment on Path Loss in Body Area Networks

**DOI:** 10.3390/s141019551

**Published:** 2014-10-20

**Authors:** Sławomir Hausman, Łukasz Januszkiewicz

**Affiliations:** Institute of Electronics, Lodz University of Technology, Wolczanska 211/215, PL 90-924, Lodz, Poland; E-Mail: slawomir.hausman@p.lodz.pl

**Keywords:** Body Area Networks, FDTD, body phantoms, on-body sensor network, wearable antennas

## Abstract

In this paper the influence of an example indoor environment on narrowband radio channel path loss for body area networks operating around 2.4 GHz is investigated using computer simulations and on-site measurements. In contrast to other similar studies, the simulation model included both a numerical human body phantom and its environment—room walls, floor and ceiling. As an example, radio signal attenuation between two different configurations of transceivers with dipole antennas placed in a direct vicinity of a human body (on-body scenario) is analyzed by computer simulations for several types of reflecting environments. In the analyzed case the propagation environments comprised a human body and office room walls. As a reference environment for comparison, free space with only a conducting ground plane, modelling a steel mesh reinforced concrete floor, was chosen. The transmitting and receiving antennas were placed in two on-body configurations chest–back and chest–arm. Path loss *vs.* frequency simulation results obtained using Finite Difference Time Domain (FDTD) method and a multi-tissue anthropomorphic phantom were compared to results of measurements taken with a vector network analyzer with a human subject located in an average-size empty cuboidal office room. A comparison of path loss values in different environments variants gives some qualitative and quantitative insight into the adequacy of simplified indoor environment model for the indoor body area network channel representation.

## 1. Body Area Network Propagation Channel

Wireless Body Area Networks (BANs) use radio transmitters which can be located inside of the human body (implants), on the body, or in its proximity. This implies at least three corresponding possible communication scenarios: in-body, on-body, and off-body. The on-body scenario includes transceivers located either directly on the human body or embedded in clothes. The off-body scenario covers the systems in which the communication takes place between transceivers located on-body and in a near range around the person (depending on the case the distance can vary from meters to even hundreds of meters). In-body communication allows the transmission of data between the devices that are located inside the body and in its proximity. This case encompasses mainly wireless endoscopes and heart pacemakers. Readers interested in a general characteristics of these communication scenarios may for instance refer to [1]. This paper will only concentrate on the on-body scenario, where antennas of the communicating devices are located either directly on the human body or in its direct vicinity at distances in the range of millimeters.

BAN systems also differ by the physical-layer channel type, which can be narrowband or ultra-wideband. Galvanic coupling may also be used instead of electromagnetic waves—where electrodes positioned on the skin replace antennas. In the paper, an Industrial Scientific Medical (ISM) 2.4 GHz band narrowband indoor channel is considered, which is used in many contemporary medical applications, such as wireless physiological sensor networks. BAN radio signal transmission is strongly affected by the presence of the human body which is a lossy medium with a complex structure, comprising tissues that have various values of dielectric permittivity and conductivity. When the radio wavelength is considerably greater than the cross-section of the body the resultant attenuation is low, however it becomes significant at gigahertz frequencies. In that case choosing radio propagation channel models for BAN is difficult because expected values of path loss through the body are high (up to about 90 dB) and may be critical for the proper operation of the network [2,3]. An in-depth simulation based analysis of the coupling between body mounted antennas using computational electrodynamics methods, such as the finite difference time domain (FDTD) method and numerical phantoms, can be found in literature, e.g., [4–7]. However, some of these considerations focus on an unrealistic case of BAN operating in free space and the influence of reflecting objects in the environment around the human body is neglected [6,7]. An interesting but specific simulation scenario is described in [5], where body communication channel in a hospital environment is considered with focus on subject-specific body mass index influence. Experience shows that for some antenna configurations (for example chest to back) it can be expected that the relatively short direct propagation path through the body introduces greater attenuation to the signal than the indoor multipath formed by the reflecting environment (floor, walls, ceiling and other objects) surrounding the BAN. Some research results provide insight into selected factors that affect propagation for body-area networks, for instance, in [8] effects of earth ground is considered. In [9] a comprehensive statistical characterization of body-to-body communication channel in a complex fire and rescue indoor training environment has been presented. In [10,11] a FDTD body channel model and a Geometrically Based Statistical Channel indoor multipath environment model are separately considered first and then combined into an indoor BAN deterministic/statistical channel model. This approach assumes independence of the two models. Also intricate analytical models for free-space BAN, for instance applying dyadic Green's function have been elaborated [12]. Some channel models are based solely on a statistical processing of measurement results [13].

**Figure 1.**
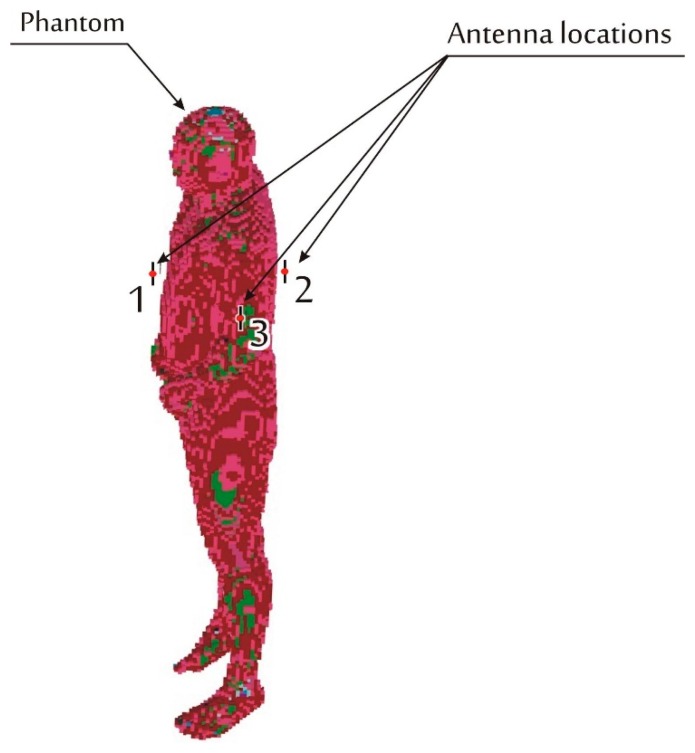
Graphical representation of the NMR Hershey body phantom in Remcom XFdtd^®^ (10 mm voxels) and antenna location: 1–chest, 2–back, 3–arm.

In contrast to those research concepts, in order to gain more quantitative insight into the influence of the reflecting multipath environment and its particular elements, an all-in-one numerical FDTD model was developed. The goal of this research was to find a simplified model of the complex building structure that is capable of giving simulation results useful for indoor BAN designers. Thus the number of obstacles (walls and ceiling) that should be included in the model was analyzed. The model consists of both a human body phantom with a pair of transmitting and receiving antennas and several examples of the surrounding multipath environment, including walls, ceiling, and floor. In this investigation the ISM 2.4 GHz band is considered. This is a remarkably important band for medical applications. Its proper use requires a reliable channel model for the radio link budget analysis, important not only for transmission reliability but also for radio node energy consumption optimization. Very intense use of the ISM 2.4 GHz frequency range by other systems also calls for interference analysis (electromagnetic compatibility) in BAN, which also requires a reliable propagation channel model. In the paper, the properties of BAN radio channel are considered as a function of link attenuation *vs.* frequency for the radio link between the transmitting and the receiving antennas located on the body in two different configurations following from three antenna locations shown in Figure 1.

## 2. Computer Simulations

The simulations of the indoor BAN channel were carried out with the use of Remcom XFdtd^®^ [14] package that implements the finite-difference time-domain (FDTD) method for electromagnetic analysis. With the BioPro^®^ [14] module the simulation can use the NMR Hershey heterogeneous model of the human body. The model is available with different degrees of discretization of tissues allowing the selection of voxel size from 1 to 10 mm. For further analysis the 10 mm model was used, as it combines a reasonable accuracy of human body representation with an acceptable amount of computer memory occupancy that is required to run simulations on the server available to the authors for relatively large simulation domain including office room walls. The simulations were performed on a PC computer equipped with two nVIDIA Tesla C2075 GPU cards. To benefit from massively multi-thread calculations that can be performed on Tesla cards, the model size had to be limited in such a way that it can fit into the RAM memory available on the cards. The model used for further analysis fits the cuboid (simulation domain) of the following dimensions: 5 m × 5.5 m × 2.52 m which resulted in a ten-fold reduction of the simulation time compared to the simulations on a 4-core CPU. In Figure 1 the body model is presented along with the location of the transmitter and receiver antennas. Both are half wave dipole antennas shortened for resonance at 2.45 GHz. The transmitter was located on the human body (phantom) chest (position 1 in Figure 1) and the receiver was placed on the back of the torso (position 2) and in the second case on the arm (position 3).

**Figure 2.**
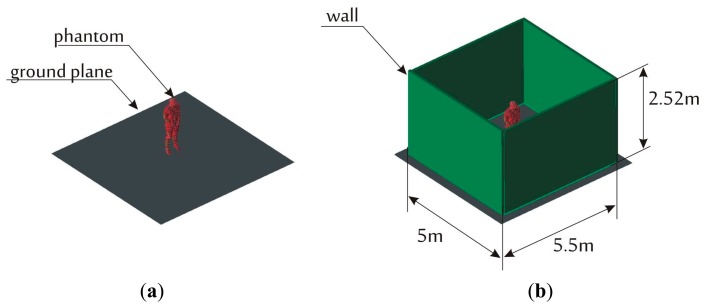
Human body model in two environments: (**a**) with a ground plane only; (**b**) with a ground plane and four walls. The third case with a ceiling is not shown but was also implemented.

Radio link path loss (signal attenuation between the antennas) for 2.2–2.8 GHz frequency range was determined for all simulation scenarios. The simulations were performed for three environments in which human body model was located. In Figure 2 the environment that consists of a conducting ground plane (modelling a floor reinforced with a steel mesh) is presented (Figure 2a) as well as an environment with a ground plane with walls (Figure 2b).

As a compromise between maximum FDTD simulation domain size (limited by memory available to the nVIDIA Tesla cards) and the accessibility of office rooms that could be used for experimental verification of the model, the size of the environment was set up as the cuboid of dimensions: 5 m × 5.5 m × 2.52 m. The thickness of the wall is in all simulations is 10 cm, typical of partition walls. The parameters of gypsum walls at 2.4 GHz were taken after [15]: ε = 1.5, loss tangent tg δ = 0.01, width *w* = 0.01 m, while the floor and ceiling are represented by a homogenous imperfect conductor with conductivity σ = 1.39·10^6^ (construction steel).

**Figure 3.**
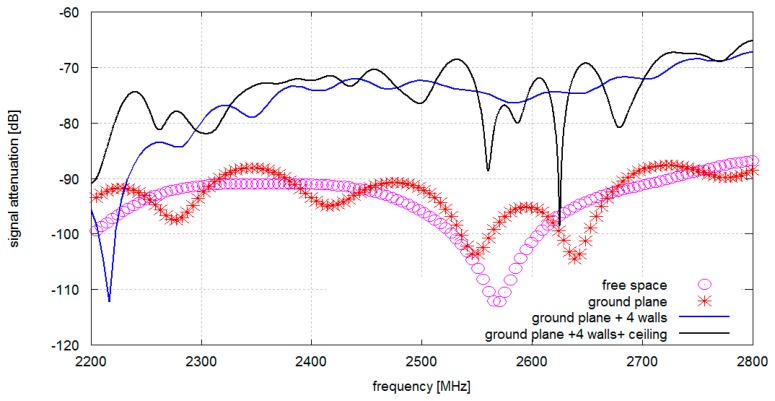
Simulated signal attenuation *vs.* frequency: A pair of antennas located on the chest and on the back (1–2) simulated for different propagation environments comprising gypsum walls.

In Figure 3 signal attenuation *vs.* frequency plots are shown for antennas located on the chest and the back of the human body for different propagation environments (presented in Figure 2). It can be observed that in the free space model there is one strong, −115 dB null at 2.55 GHz. It is likely that it corresponds to the destructive superposition of the components that get across and around the body (direct and diffracted rays). Adding a conductive floor causes multiple nulls of 10 dB depth.

When walls are added the average level of attenuation is reduced significantly, most probably by the dominating multiple reflections that provide a more effective coupling path. The minimum average attenuation is obtained where all four walls, the conductive ceiling, and the floor are present (a complete cuboid). The conductive ceiling introduced multiple nulls of significant depth which is very close to the results obtained from our experimental study.

**Figure 4.**
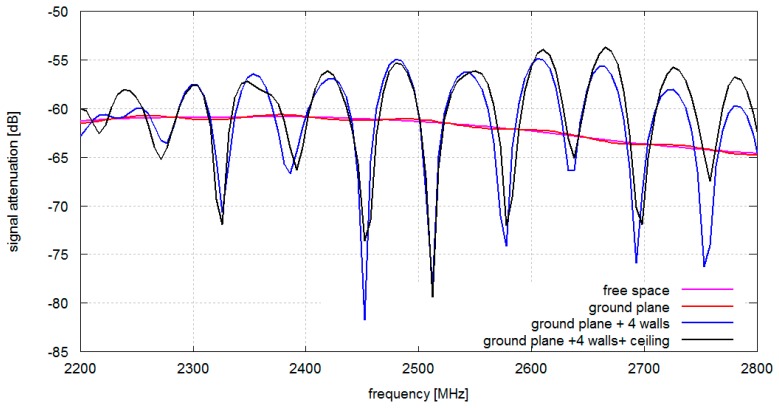
Simulated signal attenuation *vs.* frequency: A pair of antennas located on the chest and on an arm (1–3) simulated for different propagation environments comprising gypsum walls.

In Figure 4 signal attenuation between antennas located on the chest and on the arm is presented. Compared to the chest–back configuration the chest–arm configuration has stronger direct coupling between the antennas and the corresponding attenuation with ground plane only is in the range of 60 dB (compared to −90 dB for the chest–back configuration). In this case there is only a minor difference between results obtained with and without a ground plane as direct coupling dominates in the propagation mechanism. Adding walls results in a strong frequency-selective attenuation due to multipath effects. It is also worth noticing that the ceiling (modeled as a conducting structure) introduces only a small contribution to the total attenuation in the considered configuration of antennas.

## 3. Experimental Verification

**Figure 5.**
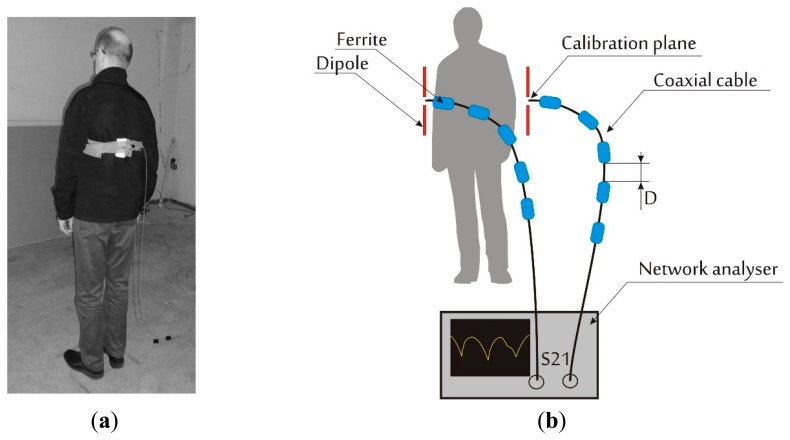
Path loss experimental setup in an empty office room with a human subject: (**a**) human subject with antennas; (**b**) setup including ferrite beads on coaxial cables to reduce the influence of the cable outer conductor radiation.

To verify the simulation results, radio link path loss (signal attenuation between the antennas) for the 2.2–2.8 GHz frequency range was measured for a scenario similar to that in simulations scenarios. The measurements were made in an empty room with geometry and dimensions similar to that used in computer simulations. The side walls were made of gypsum and the floor and ceiling of steel reinforced concrete. The measurement setup is presented in Figure 5.

Two half-wave dipole antennas were placed on the body of the human subject in positions corresponding to the simulation scenarios. The antennas were dipoles shortened for resonance at 2.45 GHz. Rohde Schwarz ZVB-14 vector network analyzer was used to measure the signal attenuation between antennas *vs.* frequency. The ferrite beads were fitted on the measurement cables to reduce radiation from the cables that might decrease the isolation between the antennas and modify their radiation pattern. The distance between the ferrites was a quarter of the wavelength for the center of the measurement band, *D* = 30 mm (see Figure 5b).

**Figure 6.**
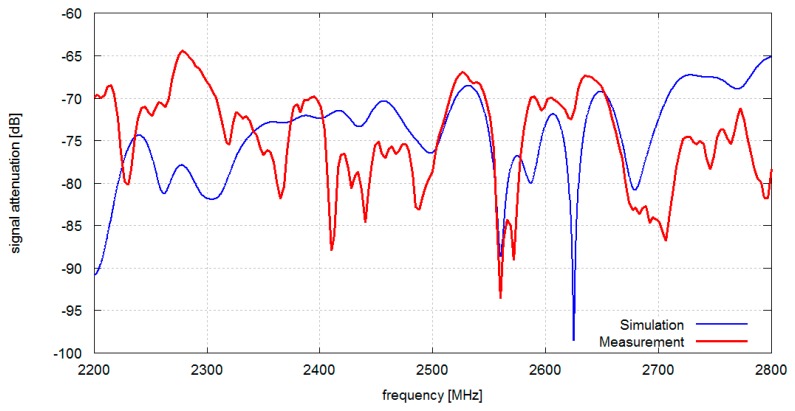
Measured and simulated signal attenuation *vs.* frequency in the indoor environment: A pair of antennas located on the chest and on the back (1–2).

**Figure 7.**
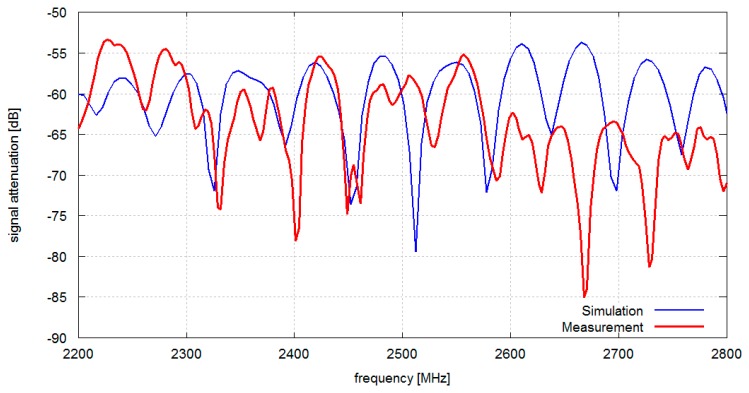
Measured and simulated signal attenuation *vs.* frequency in the indoor environment: A pair of antennas located on the chest and on an arm (1–3).

In Figure 6 the results obtained from measurements and simulations of signal attenuation between antennas located on chest and on the back (position 1–2 in Figure 1) are presented. In this case the median value of measured signal attenuation was −74.2 dB and the median value of simulated signal attenuation was −73.1 dB. In Figure 7 the results obtained for measurement and simulation of signal attenuation between antennas located on chest and on an arm (position 1–3 in Figure 1) are presented. In this case the median value of measured signal attenuation was −63.6 dB and the median value of simulated signal attenuation was −58.9 dB.

Although the location of attenuation nulls differs between simulations and measurements, the median values exhibit good agreement. Differences between simulation and measurement attenuation curves shape can be attributed to an imprecise room geometry representation in computer models, only approximated values of wall material electromagnetic properties, as well as inhomogeneity of the real building walls and ceilings.

## 4. Conclusions

In the paper the influence of the indoor environment on the signal attenuation in body area networks was studied for several simple antenna and wall geometries in the 2.4 GHz ISM band (and beyond, *i.e.*, 2.2–2.8 GHz). Both measured and simulated attenuation median values (needed e.g., to calculate the fade margin and RF power budget used in the design of BAN networks) and channel frequency responses are shown and compared. Simulations were carried out with XFdtd^®^ software package and NMR Hershey numerical human body phantom. In contrast to many other similar studies, the FDTD simulation domain included both a numerical body phantom and the surrounding room walls, floor and ceiling which is more realistic than free-space approach to BAN channel modeling.

Comparison of estimated path loss values for the considered scenarios gives a qualitative and quantitative insight into the properties of a typical indoor BAN channel (as opposed to a purely theoretical, idealized free-space BAN channel). As expected, the highest attenuation can be observed for the model with the ground plane only (90 dB) and antennas located on the chest and the back of the human torso. In a reflective multipath environment with walls, a typical value of path attenuation for the same on-body antenna locations is significantly lower (60 dB for ground plane and two walls). For the antenna pair located on the chest and on one arm the path loss is lower than for the antennas located on the opposite sides of the torso (chest and back). In this case a reflective environment also decreases attenuation of radio signal but, additionally, strong frequency selective fades can be observed. To summarize, the following main conclusions of practical (engineering) importance can be drawn from the results of measurements and numerical simulation of the indoor BAN propagation on-body scenarios in 2.4 GHz band:

1. Free-space modeling of the coupling between BAN antennas located on the human body can be applied to real indoor scenarios only for the antennas located close one to another, e.g., in the arm–chest configuration, where direct coupling between the antennas prevails over the room multipath. However in such a case free space model can be used only to approximate the median value of attenuation over the bandwidth. Frequency selective fades will not be represented.
2. For some antenna pair locations e.g., chest–back, where free-space approach (mostly presented in the literature) shows strong attenuation through the body (even up to 90 dB) in typical indoor environment the attenuation is significantly decreased. In the studied case the difference was up to 30 dB. The propagation mechanism in such a case can be dominated by room multipath propagation. In these cases BAN channel models for engineering applications should include multipath effects–even only to obtain median attenuation values.
3. Multipath effects can be easily simulated with simple room model comprising only two conducting surfaces (floor and ceiling) and dielectric walls. Such a simplification can free indoor BAN designers from creating very complex indoor environment models of target buildings. These buildings have mechanical structures that might be unknown to the BAN designer. For instance hospital rooms that are located on the same floor can differ significantly in the location of wiring and pipelines. This simplified model can be created in any full wave modeling tool. The median values of signal attenuation obtained with simulation and measurements as well as the character of frequency dependent fades (depth and frequency separation) are in good agreement. For the case of antennas located on chest and back, the corresponding simulated and measured median values differ only by 1.1 dB. For the case of antennas located on the arm and the chest the difference was 4.7 dB. Some differences between the shapes of simulation and measurement attenuation curves (e.g., location of the fades) can be attributed to an imprecise representation of room geometry and material parameters in computer models.

Indoor BAN simulation studies for a simulation domain corresponding to an average-sized office room are feasible using XFDTD software package with only a moderate PC computer configuration containing two nVIDIA Tesla C2075 GPU cards. With this computer configuration the simulations took from 45 min to 70 min depending on the FDTD algorithm convergence for a given simulated case.

The authors plan to carry out an in-depth study to formulate a practical indoor BAN radio channel path loss model taking into consideration various on-body antenna configurations as well as room dimensions and human body location as parameters. The model should be of help to engineers designing BAN destined for operation in particular indoor environments.
